# The Association between KIF6 Single Nucleotide Polymorphism rs20455 and Serum Lipids in Filipino-American Women

**DOI:** 10.1155/2014/328954

**Published:** 2014-01-23

**Authors:** Irma B. Ancheta, Cynthia A. Battie, Dan Richard, Christine V. Ancheta, Nancy Borja-Hart, Annabelle S. Volgman, Yvette Conley

**Affiliations:** ^1^School of Nursing, University of North Florida, 1 UNF Drive, Jacksonville, FL 32224, USA; ^2^University of South Florida, Tampa, FL 33612, USA; ^3^East Coast Institute for Research, Jacksonville, FL 32223, USA; ^4^Rush University Medical Center, Chicago, IL 60612, USA; ^5^University Pittsburgh, Pittsburgh, PA 15213, USA

## Abstract

The Trp719Arg allele of KIF6 rs20455, a putative risk factor for CHD especially in those with elevated low-density lipoprotein cholesterol (LDL-C), was investigated in Filipino-American women (FAW, *n* = 235) participating in health screenings in four cities. The rs20455 genotype of each subject was determined by a multiplex assay using a Luminex-OLA procedure. The risk allele Trp719Arg was present in 77% of the subjects. The genotype distribution was 23% Trp/Trp, 51% Arg/Trp, and 26% Arg/Arg. Genotype did not predict the presence of CHD risk factors. Moreover, LDL-C, HDL-C, and triglycerides mean values did not vary as a function of genotype. However, those with the Arg/Arg genotype on statin medication exhibited a significantly higher mean triglycerides level (*P* < 0.01). Approximately 60% of participants regardless of genotype exhibited LDL-C levels ≥100 mg/dL but were not taking medication. Approximately 43% of those with the Trp719Arg risk allele on statins exhibited elevated LDL-C levels. Our study suggests that the Trp719Arg allele of KIF 6 rs20455 is common among Filipino-American women; thus, even with borderline LDL-C levels would benefit from statin treatment. Secondly, many participants did not exhibit guideline recommended LDL-C levels including many who were on statin drugs.

## 1. Introduction 

Coronary heart disease is a multifactorial and complex disease resulting from the interaction of genes and environmental factors [1–5]. Genetics plays an important role in determining the inherent CHD vulnerability and in determining how a person responds to statin therapy. KIF6 is a member of the kinesin family, a class of motor proteins that are involved in the intracellular transport of membrane organelles, messenger RNA's and other protein complexes along microtubules [6–10]. Several studies focusing on Caucasians have reported that the Trp719Arg allele of single nucleotide polymorphism (SNP) rs20455 in the *KIF6* gene is associated with CHD and that carriers who carry one or two copies of the risk allele respond better to statin therapy than noncarriers [10–15]. The prevalence among Caucasians is about 59% and the risk ratio has been stated to be 1.22 (95% confidence intervals 1.12–1.32) [15]. This association with increased risk was present in African Americans, who have very high numbers (~90%) with the risk allele [15]. Chinese and Japanese also have been reported to have a high frequency (~70%) of the risk allele [15]. Other investigators were unable to detect the association of the rs20455 allele with increased CHD risk [16] even with a large sample size [17]. Ference et al. [18] carried out a large meta-analysis with 88,535 subjects in 8 randomized statin trials examining the role of KIF6 Trp719Arg allele in CHD and concluded that the risk allele increases the vulnerability to elevated LDL-C cholesterol. Thus, the KIF6 genetic variant may be a predictor of CHD in those who have elevated LDL-C. Indeed, carriers of the Trp719Argallele may have a greater increase in CHD risk per unit increase in LDL and a greater reduction in CHD risk per unit decrease in LDL, compared to noncarriers [18]. Indeed, the number needed to treat with statins to prevent a single CHD event ranged from 10 to 20 for the Trp719Arg carriers compared to >80 for noncarriers in a large meta-analysis [15]. Furthermore, the results of Ference et al. [18] suggest that the differential vulnerability to LDL-C based upon genotype may explain the reason for the prior disagreement in studies.

To the best of our knowledge despite a plethora of evidence, no studies of KIF6 Trp719Arg allele have been conducted in the Filipino population. Filipinos are the second largest minority population of Asians in America [19] and have the highest prevalence of hypertension [20], type 2 diabetes [21, 22], and metabolic syndrome [23] compared with other Asian subgroups. The heterogeneity of CHD risk factors among Asians is well established [24] and the allelic frequency distribution of rs20455 has been shown to vary across populations [15]. Scientific gaps still exist regarding the role of genetics in the predisposition to CHD in various populations. Since the rs20455 variant has not been studied in those of Filipino descent, we investigated its prevalence in association with plasma lipid levels in a cohort of Filipino American women, who attended community-based health screenings at various locations throughout the USA.

## 2. Materials and Methods

### 2.1. Study Design, Population, and Recruitment Procedure

Approval of the University Institutional Research Board (IRB) was obtained prior to the conduct of this study. This study is a descriptive, cross-sectional study of Filipino American women (*n* = 234), who participated in a cardiovascular health screening at their places of worship or cultural centers during 2011–2013. Subjects were between 40 and 65 years of age. Health screenings were performed in various US cities: Jacksonville, FL; Chicago, IL; Tampa, FL, and San Francisco, CA. In order to recruit a wide variety of subjects, advertisements were posted in church bulletins and at community organizations and stores frequently visited by Filipino women. These flyers specified inclusion and exclusion criteria and the need to be fasting for at least 12 hours on the day of the study. Participants were instructed to bring all prescription and nonprescription medications to the study site.

Participants were enrolled if they were women, self-identified as Filipino by ethnicity, agreed to volunteer for the study, and were fasting for 12 hours. Women who had severe arthritis, any autoimmune disorder, and a recent cancer diagnosis and/or presented with any infection or severe inflammation were excluded from the study important for the measurement of inflammatory markers. Informed consent was obtained after the study purpose was carefully explained. Prior to giving consent, participants were given ample time to ask questions or state concerns regarding the study. Total time for participation in the study was approximately 45 min per participant.

### 2.2. Screening Protocol

A demographic and clinical information questionnaire, including participants' medical history and current medications, was completed for each subject by trained research staff. The clinical information questionnaire included family history, smoking history, participant's medical history, and current medications. Upon completion of the survey, blood pressure was obtained using a standard Omron digital HEM-705CP sphygmomanometer on the nondominant arm, after the participant had been seated for 10 min. Measurements were repeated three times with 5 min in between each reading. Subsequently, weight and height were measured using a standard Tanita weighing scale (WB-3000). Waist circumference was measured using a tension tape guided by the NHANES measurement protocol. Lastly, a licensed phlebotomist drew 5 mL of blood via venipuncture from each participant for a series of tests including the standard lipid panel, hemoglobin A1C, serum glucose, a cardiovascular inflammatory biomarker (high-sensitivity C-reactive protein or hs-CRP), and genetic assays. The blood was captured in three test tubes containing EDTA. Sample tubes were labeled with numbers only, centrifuged, and immediately frozen. At the end of each screening session, frozen samples were sent to a CLIA certified laboratory where assays were performed using standard clinical protocols. The lipid panel assay included total cholesterol, triglycerides, high-density lipoprotein (HDL), and low-density lipoprotein (LDL-C) (Roche Modular methodology) performed at Berkeley Heartlab, Inc. (Alameda, CA). Plasma hs-CRP concentration was determined using an automated immunoturbidimetric assay (Roche Modular methodology, Berkeley Heartlab Inc.).

### 2.3. Cardiovascular Risk Factors

The operational definition for CHD risk factors for the study was based on the following guidelines. For blood pressure values, the American Heart Association and the Joint National Committee for the Prevention, Detection, Evaluation, and Treatment of High Blood Pressure (JNC 7) guidelines for the normal range of blood pressure were used (≥120 mmHg systolic and/or diastolic blood pressure of ≤80 mmHg). We used the criteria of the World Health Organization regarding cut-off for the body mass index (BMI). The National Cholesterol Education Program (NCEP) Adult Treatment Panel III guidelines were used to define the cholesterol normal reference values, namely, total cholesterol (TC) ≥200 mg/dL, triglycerides (TGL) ≥150 mg/dL, high-density lipoproteins cholesterol (HDL-C) ≤50 mg/dL, low-density lipoprotein cholesterol (LDL-C) ≥100 mg/dL, fasting blood glucose (FBG) ≥100 mg/dL (5.6 mmol/L), and waist circumference ≥35 inches (88 cm). The American Diabetes Association criteria for diabetes were used: hemoglobin A1C ≥6.5% and fasting plasma glucose ≥126 mg/dL. Metabolic syndrome was defined using both the International Diabetes federation and the National Cholesterol Education Program-Adult Treatment Panel III criteria.

### 2.4. Genetic Analysis

Celera research reagents were used to genotype the rs20455 SNP of each subject in a single-tube assay using a Luminex-OLA procedure as described previously [25]. The procedure included amplification of genomic DNA (~3 ng) by multiplex PCR followed by multiplex OLA. The resulting ligation products were hybridized to Luminex xMAP microspheres and labeled by the reporter molecule SA-PE. The xMAP microspheres were analyzed on a Luminex 100 or a Luminex 200 systems. Genotypes were subsequently determined using the Celera allele calling software as described in Iannone et al. [25].

### 2.5. Data Analysis

A confidential, password-secured database was used for data entry, management, and analysis. Inconsistencies were checked, and the data descriptions were verified by the principal investigator and the statistician. Data were analyzed using the Statistical Package for the Social Sciences (SPSS, version 19) and GraphPad Prism 5 software. Means ± standard deviations were determined for all continuous variables and number and percentage were determined for categorical variables. Analysis of variance (ANOVA) was used to compare group means and multiple stepwise linear regression determined whether risk alleles significantly predicted CHD risk factors, controlling for alternative predictors of CHD risks. Dichotomous variables were analyzed using Fisher's chi-square tests for independence with Yates continuity correction. Statistical significance was set at *P* < 0.05.

## 3. Results

### 3.1. Demographic Characteristics

Demographic characteristics of the nonrelated Filipino-American women (*n* = 234) enrolled in the study are shown in Table 1. The mean age of the women was 55.4 ± 7.1 years old and the majority (98%) were born in the Philippines with the mean length of residency in USA of 24.1 ± 13.1 years. The mean age upon arrival in the USA was 31.1 ± 10.9 years. The majority (75%) were married and many had completed a college degree and were employed in health-related professions. Most of the participants (88%) had some sort of insurance including private insurance (49%).

### 3.2. Cardiovascular Risk Factors

Clinical and morphometric measurements are shown in Table 2. Participants had a mean height of 5′1′′ and mean weight of 151 lbs. and 50% of participants were considered either overweight or obese as indicated by BMI. The majority were classified as prehypertensive because blood pressure was over the criterion set forth by the JNC 7. Approximately 36% of the women had diabetes as indicated by the levels of hemoglobin A1C. A high percentage (61%) of the participants had elevated LDL-C but the percent of the group (21%) with unhealthy HDL and elevated triglycerides (19%) was much smaller. Elevated hs-CRP was seen in 13% of the group. Approximately 50% of participants were classified as having metabolic syndrome. About 50% of participants self-reported a family history of heart disease, indicated by stroke and/or CHD events of parents and immediate siblings. Only 3% of the participants smoked and thus smoking was not thought to be a confounder in this study.

### 3.3. KIF6 rs20455 Genotype and CHD Risk Factors

The distribution of genotypes is as follows: 23% AA (Trp/Trp), 51% AG (Arg/Trp), and 26% GG (Arg/Arg). Thus, a majority (77%) of participants have at least one copy of the risk allele. This SNP was found to be in Hardy Weinberg equilibrium. There were no differences in mean age, BMI, waist circumference, hs-CRP, or HbA1c between the groups (Table 3).

Differences in lipid risk factors as a function of the rs20455 genotype and statin use are shown in Table 3. The mean value of TG was significantly elevated in the GG genotype (Arg/Arg) group (*P* < 0.009) but further analysis indicated that this elevation was only seen in those of this genotype taking statin medication. Multiple regression analyses confirmed a lack of association of genotype with TG levels but indicated a strong association with diabetes (*P* = 0.0008) indicated by HbA1c (Table 4). The prevalence of high TG (≥150 mg/dL) as a function of genotype was 10% of AA (Trp/Trp), 16% of AG (Trp/Arg), and 38% of GG (Arg/Arg) genotype (Fisher's exact *P* < 0.001) indicating that those subjects with the GG (Arg/Arg) genotype were more likely to have elevated TG levels. Similar to TG, there was a lack of association of genotype with HDL-C levels but an association with diabetes (*P* = 0.001). No differences in mean values of HDL-C between groups were apparent. No association of LDL-C with genotype nor differences in mean values were seen as a function of genotype or statin usage but an association of LDL-C with age was seen (*P* = 0.02).

We also investigated the prevalence of elevated LDL-C as a function of genotype and statin use. Many participants (60–65%; Table 5) had elevated LDL-C and were not being treated with statins including 65% of those with the Trp719Arg allele. Moreover, many in each genotype who were on statin medication exhibited elevated LDL-C including 43% of those with the Trp719Arg allele.

## 4. Discussion

The major finding of this study is a high prevalence (0.70) of the Trp719Arg allele in the Filipino-American women participants. Moreover, a high percent of women with the risk allele and elevated LDL-C levels were not being treated with statin medications and a significant number of those with the risk allele on statin medications still exhibited elevated LDL-C levels. Results from the current study are novel since an extensive search in the literature revealed no studies describing the prevalence of rs20455 SNP of KIF6 and its association with lipid levels in Filipino-American women. Our study may be the first in which the CVD rs20455 risk allele and its association to LDL-C were determined in this group of understudied minority women. Our current results showed that the majority of the FAW participants carried a single (heterozygote) or double (homozygote) copy of the risk allele. The carrier frequency seen for Filipino-American women is similar to those reported by Li et al. [15] for Asians (Japanese and Chinese) which was 72% based upon HapMap and Celera data. Thus, the carrier frequency may be higher in Asians than Caucasians who had a prevalence of 59% in one study [15]. Peng et al. [9] have reported differences in the rs20455 allele frequencies across populations.

Another finding of our study is that TG levels were significantly elevated in individuals with the Arg/Arg genotype and on statin medication. This result could be interpreted as those with Arg/Arg genotype have higher TG levels when they have elevated LDL-C but, given the small subject number, further study is warranted. Wu et al. [10] in a case-control study evaluated the association of KIF6 rs20455 SNP with angiographic CAD and serum lipid levels in the Han Chinese population of northern China. Their results demonstrated no significant differences in genotype and allele frequency between the cases (angiographic CAD) and controls (*P* > 0.05). However, further analysis showed that nonfatal MI risk and TG levels were significantly higher in 719Arg carriers compared to noncarriers (*P* < 0.05) [10].

Our findings demonstrated that there were no differences in HDL or LDL-C levels as a function of genotype; however, this finding must be viewed with caution because the sample size may not be sufficiently powered to discern an association of genotype with lipid levels. Importantly, there were a significant number of Filipino-American women, including many with the Trp719Arg allele, who had elevated LDL-C but were not being treated with statins. Others were being treated but had not achieved recommended levels of LDL-C. Although studies regarding KIF6's polymorphism and risk for CHD are equivocal, the KIF6 genetic variant may be a predictor of CHD in those who have elevated LDL-C as described in the Introduction [10–18]. Given that the number needed to treat with statins to prevent a single CHD event ranged from 10 to 20 for the Trp719Arg carriers, it is imperative that those with this allele be treated for elevated LDL-C [15]. The elevated LDL-C in participants on statin medication could indicate noncompliance with statin medications due to muscle pains or other side effects of the drugs; however, we did not capture data about nonadherence to prescribed cholesterol-lowering medication [26, 27]. Additionally, elevated LDL-C levels despite use of statins in this population may be conceivably due to genetic differences of this population [9]. These results may also be due to the differential performance of lipophilic and hydrophilic statins [28, 29]. Bonsu et al. [28] conducted a systematic review and meta-analysis of lipophilic and hydrophilic statins on heart failure patients. The investigators claimed that there are differences in the pleotropic effects of statin medications due to differences in lipophilicity in the type of statin medications. Lipophilic statins utilize passive diffusion for entering cells while hydrophilic statins use carrier-mediated process for uptake in the liver cells. Although there are controversial outcomes on the effects of lipophilic statins, this group of statins appear to show more beneficial effects than those of hydrophilic statins, though improved outcomes have been reported for rosuvastatin in the Controlled Rosuvastatin in Multinational Trial in Heart Failure (CORONA) and the Gruppo Italiano per lo Studio della Sopravvivenza nell'Infarto Miocardico Heart Failure (GISSI-HF) trials [29]. Regardless of cause, further research is required to determine why LDL-C is not at optimal levels among Filipino-American women even when taking cholesterol-lowering medications. Our study indicates that this population needs to be more carefully monitored and treated for elevated LDL-C. Specific statin medications may have adverse effects in Asian populations [30]. In 2005, the US Food and Drug Administration provided additional safety label warning for rosuvastatin since a pharmacokinetic study that included a diverse population of Asians showed that rosuvastatin drug levels were found to be approximately twofold higher when compared with the Caucasian control group. This in effect may produce increased risk of muscle myopathies and risk of kidney failure in Asians [30].

Many clinical trials have shown the benefit of lowering cholesterol levels even in those with relatively normal cholesterol levels and no previous myocardial infarctions [31–33]. One clinical trial of participants with myocardial infarction and plasma total cholesterol <240 mg/dL and LDL-C of 115–174 mg/dL randomized to 40 mg of pravastatin per day or placebo had 10.2% events in the pravastatin group and 13% events in the placebo group indicating that cholesterol-lowering treatment is beneficial for CAD patients with borderline cholesterol levels [31]. Pravastatin lowered cholesterol by 20% and LDL-C by 26% and reduced MI incidence in men with hypercholesterolemia and no previous MI [32]. These studies also suggest the need for cholesterol lowering in carriers of the rs20455 allele. For example rs20455 allele carriers had a hazard ratio of 1.50 in the CARE trial [31] and odds ratio of 1.55 (95%CI 1.14–2.09) in WOSCOPS trial [32]. For the rs20455 carriers the absolute risk reduction by pravastatin was 4.89% in the CARE trial and 5.49% in the WOSCOPS trial. In the PROSPER [33] study among the elderly population with previous CVD disease on pravastatin therapy, there was an absolute risk reduction of 6.3% in KIF6 carriers versus 1.2% in noncarriers with a 33.6% reduction of relative risk among carriers with the number needed to treat with pravastatin significantly lower in carriers compared to noncarriers (16 versus 83). Thus, these three trials demonstrated that KIF6 risk allele carriers had an increased risk of developing coronary events and the use of pravastatin significantly reduced the risk. Observational studies with multiple populations also found increased risk in allele carriers [34–38], Another study of 539 participants showed that the KIF6 risk variant was associated with cardiac events with a hazard ratio of 1.33 after adjustment for age, sex race, high-sensitivity C-reactive protein, and LDL-C [34]. In the ARIC [35, 36] study, carriers of the KIF6 719Arg risk variant had a higher incidence of CHD with a hazard ratio of 1.22 after model was adjusted for age and sex. In the WHS [38] study, carriers of the KIF67 719Arg risk variant had a greater CHD risk with a hazard ratio of 1.24 and a hazard ratio of 1.34 associated with risk for MI.

In contrast, other large studies have not found an association of Kif6 allele with statin response [39, 40]. The Heart Protection Study (HPS) with 18,348 randomized patients found that there was no impact of KIF6 genotype on statin therapy and vascular events across all genotypes [39, 40]. In The JUPITER Trial, Caucasian participants with low LDL-C <130 mg/dL and elevated high-sensitivity C-reactive protein ≥2 mg/L were randomly assigned to rosuvastatin or placebo and followed for first major CVD event [12]. There were no differences in vascular event rates in KIF6 risk allele carriers compared to noncarriers (hazard ratio of 0.91) nor amount of LDL-C reduction. Differences in study design between the earlier statin trials and more recent trials such as HPS [39, 40] and JUPITER [12] could help explain the different findings. In the HPS placebo patients have been intensively treated with 40 mg simvastatin for 6 weeks prior to randomization which substantially reduced LDL-C levels and according to Ference et al. [18] could attenuate risk associated with KIF6 and therefore explain the lack of a differential response to statin therapy in those with KIF6 genotype. And, similarly, in JUPITER only participants with low LDL-C levels were enrolled in the study and were treated with high-dose rosuvastatin. Such contradictory findings necessitate further investigation of the effect of KIF6 polymorphism on cardiovascular health.

### 4.1. Limitations

Certain study limitations merit comment. First, the study was based upon a small number of subjects and the significant genetic admixture of Filipinos also presents a challenge when small population subsets are studied. The exclusion of Filipino-American men was another limitation to the study, although it should be noted that most studies include only men and women have been an understudied gender. Moreover, we have developed a successful method of recruiting women for these sorts of studies in community settings. It is not clear if this method would work for Filipino-American men.

Third, since this study was only conducted on the Filipino population in the United States, the generalizability to Filipinos residing in other countries is not known. Fourth, the use of the cross-sectional design limits examining risk factors for heart disease at a single point in time without the ability to measure cardiovascular outcomes.

## 5. Conclusion

Our results showed that 70% of a cohort of nonrelated Filipino-American women are carriers of the Trp719Arg risk allele (rs20455 SNP of the KIF6 gene). Importantly, many of these women, even those treated with statins, did not achieve guideline recommended LDL-C levels. Although larger population studies are needed to confirm our findings, the results indicate that Filipino-American women need to be more carefully monitored and treated for elevated cholesterol in order to reduce the incidence of CHD in this population.

## Figures and Tables

**Table 1:** Demographic characteristics of the Filipino-American women subjects.

Demographic characteristics	Mean ± SD (*n* = 234)	Frequency (%)
Age	51.5 ± 7	—
No. of years in the USA	24.0 ± 13	—
Age—arrival in USA	31.0 ± 11	—
Marital status		
Single		8
Married		73
Divorced/widow		19
Philippine-born		98
Residency in the USA		
<5 years		11
5–10 years		6
10–20 years		26
Over 20 years		57
Income		
<$12,000/year		17
$13,000–$40,000		47
$41,000–$69,000		20
$70,000 and above		16
Education		
High school		18
Some college		21
4 year degree		52
Graduate degree		9
Occupation		
Health occupations		34
Healthcare insurance		
With insurance		87

**Table 2:** Morphometric measurements and cardiovascular risk factors of the Filipino-American women participants (*n* = 235).

Measurement	Means ± SD	Percent of group (risk level)
Weight (lbs.)	151 ± 22	
Height (inches)	61 ± 2	
Body mass index	29 ± 4	37 (≥25 kg/m^2^) 15 (≥30 kg/m^2^)
Waist circumference	40 ± 4	79 (≥35 inches)
Systolic blood pressure	131 ± 19	64 (≥120 mmHg)
Diastolic blood pressure	87 ± 10	61 (≤80 mmHg)
Fasting blood glucose	101 ± 25	38 (≥100 mg/dL)
Hemoglobin A1C	6.0 ± 0.8	36 (≥6.5%)
Total cholesterol	201 ± 45	44 (≥200 mg/dL)
Triglycerides	116 ± 67	19 (≥150 mg/dL)
Low-density lipoprotein-C	122 ± 35	61 (≥100 mg/dl)
High-density lipoprotein-C	62 ± 15	21 (≤50 mg/dL)
High-Sensitivity C-Reactive protein	1.97 ± 3.0	13 (<3.0 mg/L)
Metabolic syndrome		56
Smoking		3
Family history		48

Note: metabolic syndrome was defined by both the International Diabetes Federation (IDF) and the National Cholesterol Education Program—Adult Treatment Panel III (NCEP/ATP III) criteria.

**Table 3:** Selective cardiovascular disease risk factors as a function of the rs20455 genotype and statin medication usage.

Risk factors	AA Trp/Trp *n* = 53 23%	AG Arg/Trp *n* = 120 51%	GG Arg/Arg *n* = 62 26%	ANOVA *P* value
Age				
Total	52 ± 7	54 ± 7	52 ± 6.9	0.09
No statins	51 ± 10	52 ± 9	50 ± 10	0.28
Statins	52 ± 4	54 ± 6	52 ± 7	
HDL-C				
Total	60 ± 14	64 ± 15	61 ± 16	0.21
No statins	60 ± 16	65 ± 7	63 ± 15	0.06
Statins	56 ± 11	61 ± 15	52 ± 14	
LDL-C				
Total	114 ± 36	113 ± 38	113 ± 37	0.71
No statins	118 ± 39	119 ± 39	116 ± 34	0.59
Statins	106 ± 32	101 ± 36	113 ± 40	
TG				
Total	119 ± 58	109 ± 67	130 ± 74^1,2^	0.009
No statins	118 ± 60	107 ± 74	111 ± 54	
Statins	121 ± 50	113 ± 39	177 ± 97^3^	0.002
Waist Circ				
Total	37 ± 7	37 ± 6	36 ± 7	
No statins	36 ± 6	37 ± 4	37 ± 4	0.93
Statins	37 ± 4	36 ± 7	37 ± 3	
BMI				
Total	26 ± 4	28 ± 4	26 ± 4	0.06
No statins	26 ± 4	27 ± 4	25 ± 5	
Statins	30 ± 5	30 ± 4	27 ± 5	0.15
Hs-CRP				
Total	1.41 ± 1.5	1.86 ± 2.2	1.55 ± 2.1	0.45
No statins	2.1 ± 3.9	2.2 ± 3.3	1.6 ± 1.7	
Statins	1.6 ± 2.9	1.4 ± 1.9	2.2 ± 3.0	0.87
HbA1c				
Total	5.8 ± 0.46	5.8 ± 0.49	6.01 ± 0.97	0.39
No statins	5.8 ± 0.48	5.8 ± 0.48	5.9 ± 0.66	
Statins	6.3 ± 1.2	6.1 ± 0.54	6.4 ± 1.5	0.17

Note: HDL-C: high-density lipoprotein cholesterol; LDL-C: low-density lipoprotein-cholesterol; TG: triglycerides; Waist Circ: waist circumference; BMI: body mass index; hs-CRP: high-sensitivity C-reactive protein; HbA1c: Hemoglobin A1c. ^1^ *P* < 0.05 Trp/Trp versus Arg/Arg, ^2^ *P* < 0.01 Arg/Trp versus Arg/Arg, and ^3^Arg/Arg versus all other groups *P* < 0.001; AA no statins *n* = 22, statins *n* = 12; AG no statins *n* = 48, statins *n* = 21; GG no statins *n* = 26, statins *n* = 16.

**Table 4:** Multiple regression analysis of cardiovascular risk factors.

Variables	TG T ratio (*P* value)	LDL T ratio (*P* value)	HDL T ratio (*P* value)
Genotype	0.57 (0.27)	0.18 (0.86)	1.11 (0.24)
HbA1c	3.42 (0.0008)	0.75 (0.45)	3.36 (0.001)
hs-CRP	0.97 (0.34)	0.41 (0.68)	1.92 (0.06)
Age	0.46 (0.64)	2.32 (0.02)	1.15 (0.25)
BMI	0.56 (0.57)	1.23 (0.22)	0.22 (0.86)

Note: HbA1c: hemoglobin A1c; hs-CRP: high-sensitivity C-reactive protein.

**Table 5:** Percent of participants with elevated LDL as a function of genotype and statin usage.

Medications	Trp/Trp (AA)	Arg/Trp (AG)	Arg/Arg (GG)	Arg/Trp Arg/Arg (AG + GG)	Fisher's exact test *P* Value
No statins	*n* = 22	*n* = 48	*n* = 26	*n* = 74	
% elevated LDL-C	69%	67%	62%	65%	0.41
Statins	*n* = 12	*n* = 21	*n* = 16	*n* = 37	
% elevated LDL-C	58%	33%	56%	43%	0.31

## References

[1] Cohen JC (2006). Genetic approaches to coronary heart disease. *Journal of the American College of Cardiology*.

[2] Lusis AJ, Fogelman AM, Fonarow GC (2004). Genetic basis of atherosclerosis: part II: clinical implications. *Circulation*.

[3] Gibbons GH, Liew CC, Goodarzi MO (2004). Genetic markers: progress and potential for cardiovascular disease. *Circulation*.

[4] Peden JF, Farrall M (2011). Thirty-five common variants for coronary artery disease: the fruits of much collaborative labour. *Human Molecular Genetics*.

[5] The IBC 50K CAD Consortium (2011). Large-scale gene-centric analysis identifies novel variants for coronary artery disease. *PLoS Genetics*.

[6] Hirokawa N (1998). Kinesin and dynein superfamily proteins and the mechanism of organelle transport. *Science*.

[7] Miki H, Setou M, Kaneshiro K, Hirokawa N (2001). All kinesin superfamily protein, KIF, genes in mouse and human. *Proceedings of the National Academy of Sciences of the United States of America*.

[8] Seiler S, Kirchner J, Horn C, Kallipolitou A, Woehlke G, Schliwa M (2000). Cargo binding and regulatory sites in the tail of fungal conventional kinesin. *Nature Cell Biology*.

[9] Peng P, Lian J, Huang RS (2012). Meta-analyses of KIF6 rs20455 in coronary heart disease and statin therapeutic effect. *PLoS ONE*.

[10] Wu G, Li GB, Dai B (2012). Association of KIF6 variant with lipid level and angiographic coronary artery disease events risk in the Han Chinese population. *Molecules*.

[11] Shiffman D, Chasman DI, Zee RYL (2008). A kinesin family member 6 variant is associated with coronary heart disease in the Women’s Health Study. *Journal of the American College of Cardiology*.

[12] Ridker PM, Macfadyen JG, Glynn RJ, Chasman DI (2011). Kinesin-like protein 6 (KIF6) polymorphism and the efficacy of rosuvastatin in primary prevention. *Circulation: Cardiovascular Genetics*.

[13] Shiffman D, Sabatine MS, Louie JZ (2010). Effect of pravastatin therapy on coronary events in carriers of the KIF6 719Arg allele from the cholesterol and recurrent events trial. *The American Journal of Cardiology*.

[14] Iakoubova OA, Tong CH, Rowland CM (2008). Association of the Trp719Arg polymorphism in kinesin-like protein 6 with myocardial infarction and coronary heart disease in 2 prospective trials. The CARE and WOSCOPS trials. *Journal of the American College of Cardiology*.

[15] Li Y, Iakoubova OA, Shiffman D, Devlin JJ, Forrester JS, Superko HR (2010). KIF6 polymorphism as a predictor of risk of coronary events and of clinical event reduction by statin therapy. *The American Journal of Cardiology*.

[16] Bare LA, Ruiz-Narvaéz EA, Tong CH (2010). Investigation of KIF6 Trp719Arg in a case-control study of myocardial infarction: a Costa Rican population. *PLoS ONE*.

[17] Assimes T, Holm H, Katherisan S (2010). Lack of assoaition between the rs20455 polymorphism in Kinesin-like protein-6 and coronary artery disease in 19 case-control studies. *Journal of the American College of Cardiology*.

[18] Ference BA, Yoo W, Flack JM, Clarke M (2011). A common KIF6 polymorphism increases vulnerability to low-density lipoprotein cholesterol: two meta-analyses and a meta-regression analysis. *PLoS ONE*.

[19] Larsen LJ (2004). The foreign born population in the United States: 2003. *Current Population Reports*.

[20] Ursua RA, Islam NS, Aguilar DE (2013). Predictors of hypertension among Filipino immigrants in the Northeast US. *Journal of Community Health*.

[21] Araneta MRG, Grandinetti A, Chang HK (2010). A1C and diabetes diagnosis among Filipino Americans, Japanese Americans, and Native Hawaiians. *Diabetes Care*.

[22] Holland AT, Zhao B, Wong EC, Choi SE, Wong ND, Palaniappan LP (2013). Racial/ethnic differences in control of cardiovascular risk factors among type 2 diabetes patients in an insured, ambulatory care population. *Journal of Diabetes and Its Complications*.

[23] Ancheta IB, Battie CA, Tuason T, Ancheta CV (2012). A comparison of metabolic syndrome (MetS) risk factors in Filipino women and Filipino American women: A Pilot Study. *Ethnicity & Disease*.

[24] Palaniappan LP, Araneta MR, Assimes TL (2010). Call to action: cardiovascular disease in Asian Americans: a science advisory from the American Heart Association. *Circulation*.

[25] Iannone M, Taylor J, Chen J (2000). Multiplexed single nucleotide polymorphism genotyping by oligonucleotide ligation and flow cytometry. *Cytometry*.

[26] Kim YS, Sunwoo S, Lee HR (2002). Determinants of non-compliance with lipid-lowering therapy in hyperlipidemic patients. *Pharmacoepidemiology and Drug Safety*.

[27] Peterson AM, McGhan WF (2005). Pharmacoeconomic impact of non-compliance with statins. *PharmacoEconomics*.

[28] Bonsu KO, Kadirvelu A, Reidpath DD (2013). Lipophilic versus hydrophilic statin therapy for heart failure: a protocol for an adjusted indirect comparison meta-analysis. *Systematic Reviews*.

[29] Ganesan S, Ito MK (2013). Coenzyme Q10 ameliorates the reduction in GLUT4 transporter expression induced by simvastatin in 3T3-L1 adipocytes. *Metabolic Syndrome and Related Disorders*.

[30] U. S. Food and Drug Administration (2013). *FDA Public Health Advisory for Crestor (Rosuvastatin)*.

[31] Sacks FM, Pfeffer MA, Moye LA (1996). The effect of pravastatin on coronary events after myocardial infarction in patients with average cholesterol levels. *The New England Journal of Medicine*.

[32] Shepherd J, Cobbe SM, Ford I (1995). Prevention of coronary heart disease with pravastatin in men with hypercholesterolemia. *The New England Journal of Medicine*.

[33] Shepherd J, Blauw GJ, Murphy MB (2002). Pravastatin in elderly individuals at risk of vascular disease (PROSPER): a randomised controlled trial. *The Lancet*.

[34] Cushman M, Cornell ES, Howard PR, Bovill EG, Tracy RP (1995). Laboratory methods and quality assurance in the Cardiovascular Health Study. *Clinical Chemistry*.

[35] Fried LP, Borhani NO, Enright P (1991). The Cardiovascular Health Study: design and rationale. *Annals of Epidemiology*.

[36] Szklo M, Barnes R, Folsom A (1989). The Atherosclerosis Risk in Communities (ARIC) Study: design and objectives. The ARIC investigators. *American Journal of Epidemiology*.

[37] White AD, Folsom AR, Chambless LE (1996). Community surveillance of coronary heart disease in the Atherosclerosis Risk in Communities (ARIC) Study: methods and initial two years’ experience. *Journal of Clinical Epidemiology*.

[38] Ridker PM, Cook NR, Lee I-M (2005). A randomized trial of low-dose aspirin in the primary prevention of cardiovascular disease in women. *The New England Journal of Medicine*.

[39] Hopewell JC, Parish S, Clarke R (2011). No impact of KIF6 Genotype on vascular risk and statin response among 18,348 randomized patients in the heart protection study. *Journal of the American College of Cardiology*.

[40] Hopewell JC, Parish S, Offer A (2013). Impact of common genetic variation on response to simvastatin therapy among 18 705 participants in the Heart Protection Study. *European Heart Journal*.

